# Supplementary material for: miR-665-Mediated Regulation of AHCYL2 and BVES Genes in Recurrent Implantation Failure
Source: Genes (Basel). 2024 Feb 15;15(2):244. doi: 10.3390/genes15020244 (PMC10888078; doi:10.3390/genes15020244)
Supplement: Supplementary file 1 [file genes-15-00244-s001.zip › supplementary figure S1.pptx]

## Slide 1
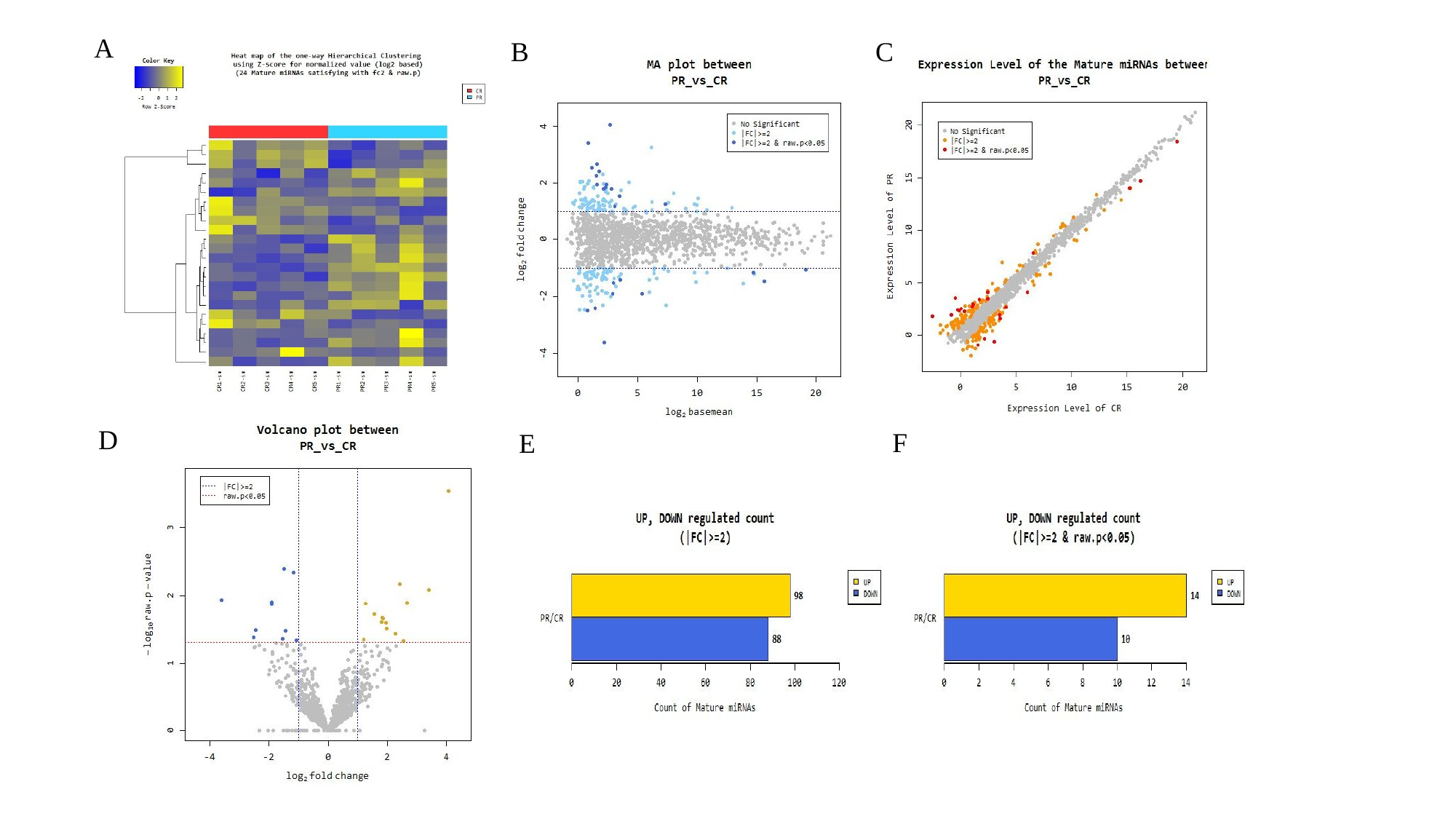

A
B
C
D
F
E

## Slide 2
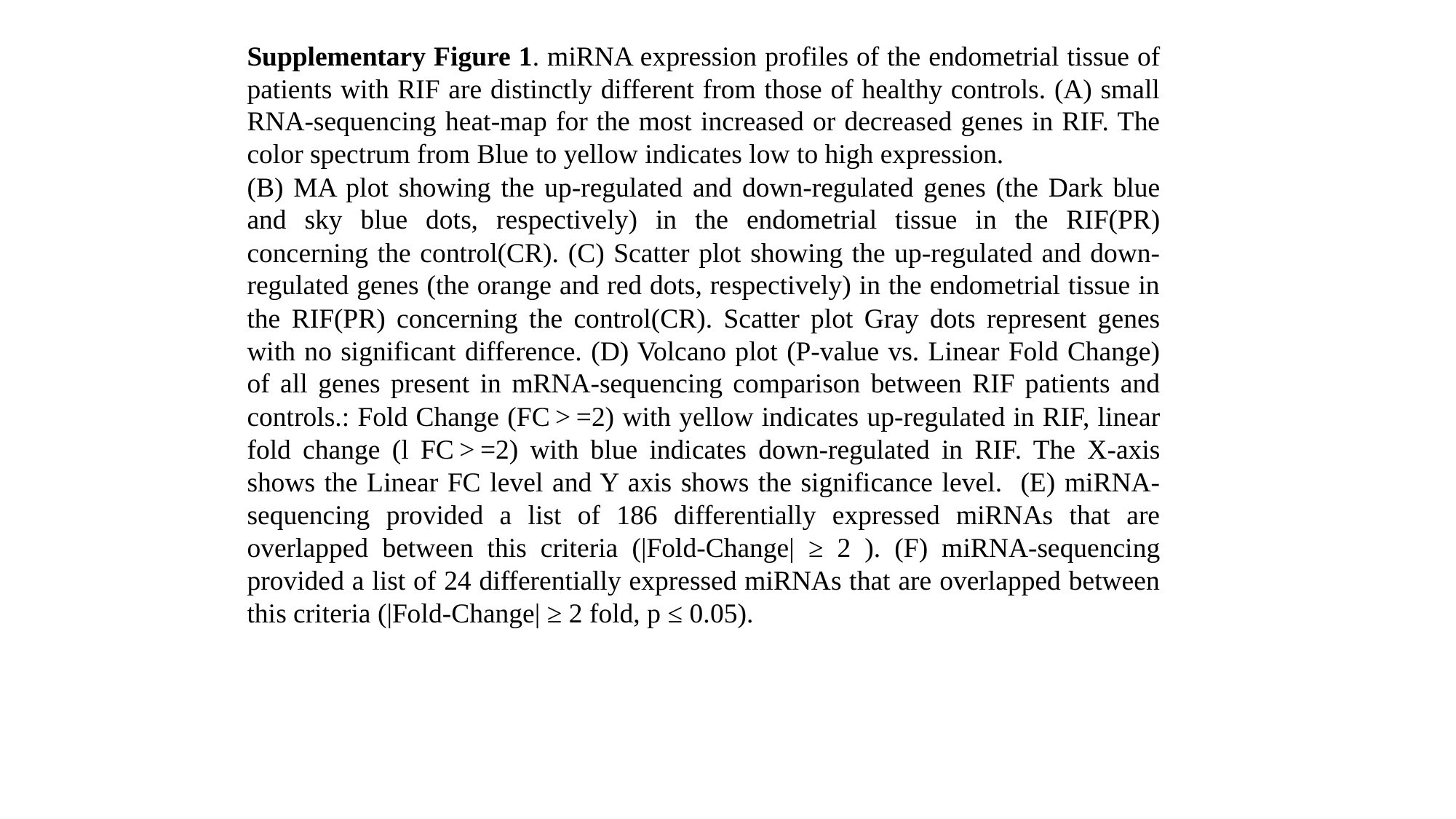

Supplementary Figure 1. miRNA expression profiles of the endometrial tissue of patients with RIF are distinctly different from those of healthy controls. (A) small RNA-sequencing heat-map for the most increased or decreased genes in RIF. The color spectrum from Blue to yellow indicates low to high expression.
(B) MA plot showing the up-regulated and down-regulated genes (the Dark blue and sky blue dots, respectively) in the endometrial tissue in the RIF(PR) concerning the control(CR). (C) Scatter plot showing the up-regulated and down-regulated genes (the orange and red dots, respectively) in the endometrial tissue in the RIF(PR) concerning the control(CR). Scatter plot Gray dots represent genes with no significant difference. (D) Volcano plot (P-value vs. Linear Fold Change) of all genes present in mRNA-sequencing comparison between RIF patients and controls.: Fold Change (FC > =2) with yellow indicates up-regulated in RIF, linear fold change (l FC > =2) with blue indicates down-regulated in RIF. The X-axis shows the Linear FC level and Y axis shows the significance level. (E) miRNA-sequencing provided a list of 186 differentially expressed miRNAs that are overlapped between this criteria (|Fold-Change| ≥ 2 ). (F) miRNA-sequencing provided a list of 24 differentially expressed miRNAs that are overlapped between this criteria (|Fold-Change| ≥ 2 fold, p ≤ 0.05).
